# MiR-219-5p suppresses cell proliferation and cell cycle progression in esophageal squamous cell carcinoma by targeting CCNA2

**DOI:** 10.1186/s11658-018-0129-6

**Published:** 2019-02-05

**Authors:** Qiang Ma

**Affiliations:** Department of Oncology, People’s Hospital of Xintai City, No. 1329 Xinfu Road, Xintai, 271200 Shandong Province China

**Keywords:** Esophageal squamous cell carcinoma, miR-219-5p, CCNA2, Cell proliferation, G2/M phase arrest

## Abstract

**Background:**

We investigated the potential regulatory role of miR-219-5p in esophageal squamous cell carcinoma (ESCC) and looked at the underlying mechanisms in ESCC.

**Methods:**

Real-time PCR was used to determine the levels of miR-219-5p in ESCC tissues and cell lines. The effects of miR-219-5p and cyclin A2 (CCNA2) on cell proliferation and cell cycle progression were evaluated using MTT, colony formation and flow cytometry assays with ESCC cell lines EC9706 and TE-9. Bioinformatics techniques and the luciferase reporter assay were applied to validate CCNA2 as the miR-219-5p target in ESCC cells. The mRNA and protein levels of CCNA2 were measured using real-time PCR and western blotting.

**Results:**

MiR-219-5p expression was significantly lower in ESCC tissues and cells than in healthy tissues. Upregulation of miR-219-5p repressed cell proliferation and induced cell cycle arrest at the G2/M phase. CCNA2 was identified and confirmed as a direct downstream target of miR-219-5p and its expression negatively correlated with miR-219-5p profiles in ESCC tissues. Knockdown of CCNA2 potentiated the effects of miR-219-5p on cell proliferation and cell cycle distribution.

**Conclusions:**

Our results demonstrate that miR-219-5p might function as a tumor suppressor by directly targeting CCNA2 expression. It could serve as a new therapeutic target for ESCC.

## Background

Esophageal squamous cell carcinoma (ESCC) is one of the most aggressive squamous cell carcinomas, generally exhibiting a high rate of lymph node metastasis and tumor invasion into adjacent tissues and organs [1]. It has one of the highest global mortality rates. ESCC is the fifth and seventh leading cause of cancer-related death in China and Japan, respectively [2, 3]. Despite considerable progress in its treatment, the prognosis for ESCC patients remains extremely poor. Studies on the underlying molecular mechanisms are essential in the search for new treatments.

Thus far, epidemiologic investigations have identified tobacco and alcohol as strong risk factors for the initiation and development of ESCC [4]. Various studies have provided a landscape of molecular events that occur during the onset and development of ESCC [5, 6].

MicroRNAs (miRNAs) are endogenous non-coding RNA molecules (21–25 nt long) [7]. They can pair with 3’UTR sites in their target mRNAs to repress their translation [8]. MiRNAs are now recognized as critical participants in a variety of physiological and pathological processes in eukaryotes [9].

MiR-219-5p is an intergenic miRNA encoded by two genomically distinct loci, one on chromosome 5, the other on chromosome 9 [10]. In recent years, miR-219-5p has been found to play a vital role in the progression of various cancers, including papillary thyroid carcinoma (PTC) [10], malignant melanoma [11], colorectal cancer [12] and hepatocellular carcinoma (HCC) [13]. Li et al. [14] showed that miR-219-5p is downregulated in gastric cancer tissues and cell lines. Overexpression of miR-219-5p could inhibit gastric cancer cell malignancy by targeting the liver receptor homolog-1/Wnt/β-catenin signaling pathways. In colorectal cancer, miR-219-5p functions as a new tumor suppressor via negative regulation of lymphoid enhancer-binding factor 1 [15]. However, the expression and biological function of miR-219-5p in ESCC remains undefined.

The cell division cycle is a complex process that is tightly controlled by coordinated action of multiple proteins, the degradation of which is guided by sequential phosphorylation–dephosphorylation events [16]. Cyclins and their catalytic partners, the cyclin-dependent kinases (CDKs), are components of the mammalian core cell cycle engine that triggers cell cycle progression [17]. In mammals, cyclin A occurs as the testis-specific gene cyclin A1 (CCNA1) and the widely expressed gene cyclin A2 (CCNA2) [18]. CCNA2 accumulates during G1 phase and functions as an essential regulator of the G1/S and G2/M transition [19]. Its expression levels have been shown to be significantly lower in various human cancers, indicating that reduced expression of CCNA2 may be associated with cancer development and growth [20].

Using the Gene Expression Omnibus database, differentially expressed profiles of CCNA2 were predicted for ESCC and normal samples, and the gene was suggested as a potential therapeutic target in ESCC [21]. However, as with miR-219-5p, the biological function of CCNA2 in ESCC remains unclear.

In this study, we determined the gene expression of miR-219-5p in ESCC tissues and cell lines, and its relationship with the biological behavior of ESCC cells. Potential targets of miR-219-5p were predicted using bioinformatics techniques, and interactions between miR-219-5p and CCNA2 were chosen for further investigation. We assessed whether regulation of CCNA2 by miR-219-5p would provide a possible therapeutic strategy for ESCC treatment.

## Materials and methods

### Human tissue samples

A total of 20 pairs of ESCC and corresponding normal esophageal tissues were collected in parallel from patients undergoing curative-intent surgery at the People’s Hospital of Xintai City. The patients’ characteristics, including sex, age, TNM stage and histological grade are summarized in Table 1. All tissues were immediately dissected, placed on ice, snap-frozen in liquid nitrogen, and stored at − 80 °C until processing. The histological sections were diagnosed by two pathologists in accordance with the *WHO Classification of Tumors of the Digestive System* (2010).

**Table 1:** Clinicopathological characteristics in esophageal squamous cell carcinoma patients (*n* = 20)

Patient characteristics	Cases (*n* = 20)
Sex	
Male	13
Female	7
Age	
< 65	12
≥ 65	8
Tumor size (cm)	
< 4	14
≥ 4	6
Histological grade	
G1	11
G2 + G3	9
TNM stage	
I/II	16
III/IV	4

*TNM* tumor node metastasis

### Cell culture and transfection

Human ESCC cell lines (KYSE150, ECA109, EC9706 and TE-9) and a normal esophageal epithelial cell line (Het-1A) were purchased from the Institute of Biochemistry and Cell Biology of the Chinese Academy of Sciences. All cell lines were cultured in RPMI-1640 medium with 10% heat-inactivated fetal bovine serum (FBS), 100 units of penicillin/ml (Sigma), and 100 mg of streptomycin/ml (Sigma) in an incubator containing 5% CO_2_ at 37 °C.

The miR-219-5p mimics (5’-UGGCAGUGUCUUAGCUGGUUGU-3′), CCNA2 small interfering RNA (si-CCNA2: 5’-GGGGTAATGCAGAAGTGAT-3′), and relative negative scramble control RNAs were synthesized at GenePharma Company. For cell transfection, EC9706 and TE-9 cells were seeded at 3 × 10^5^ cells per well in a 6-well plate and cultured overnight. Transfection was performed using Lipofectamine 2000 transfection reagent (Invitrogen) following the manufacturer’s protocols with the final concentration of 25 nM for the miR-219-5p mimics and 50 nM for si-CCNA2.

### RNA extraction and real-time PCR

Total RNA was extracted from tissues and cells using TRIzol Reagent (Invitrogen) and 2 μg total RNA was reversed transcribed into cDNA with Superscript II reverse transcriptase (Invitrogen) following the manufacturer’s instructions. The expression levels of miR-219-5p and CCNA2 mRNA were quantified using an Applied Biosystems 7300 Real-Time PCR System. The real-time PCR data were quantified according to the formula 2^−ΔΔCt^. The primer sequences were: miR-219-5p: 5’-CGGTGATTGTCCAAACGCAATTC-3′; CCNA2 forward: 5’-CAGAAAACCATTGGTCCCTC-3′ and reverse: 5’-CACTCACTGGCTTTTCATCTTC-3′; GAPDH forward: 5’-GCACCGTCAAGGCTGAGAAC-3′ and reverse: 5’-TGGTGAAGACGCCAGTGGA-3′; and U6: 5’-TGGTGAAGACGCCAGTGGA-3′. The expression levels of miR-219-5p and CCNA2 were normalized using U6 and GAPDH as the respective internal controls.

### Cell proliferation assay

Following 48 h cell transfection, cells were trypsinized, re-suspended and seeded at a density of 5 × 10^3^ cells per well in 96-well plates. At the indicated time points, 10 μl 5 mg/ml MTT reagent was added to each well and the cells were incubated for another 4 h at 37 °C. The supernatant was discarded and 200 μl of dimethylsulfoxide (DMSO) was added to each well. The absorbance at 595 nm was measured on a microplate reader (Thermo Fisher Scientific).

### Colony formation assay

After 48 h of cell transfection, a total of 3500 cells were plated in 6-well plates and continuously cultured for 15 days. After gentle washing with PBS, the cells were fixed with 4% formaldehyde for 30 min, stained for 15 min with 0.2% crystal violet solution, then air dried. The surviving colonies (≥50 cells/colony) were counted under a microscope.

### Cell cycle analysis

For cell cycle analysis, the transfected cells were seeded in 6-cm dishes at 2 × 10^5^ cells per dish and cultured until approximately 80% confluence. Then cells were harvested by trypsinization and washed with ice-cold PBS. After fixation in 75% ethanol, the cells were treated with RNase A (Sigma-Aldrich) and stained with 500 μl propidium iodide (PI; Sigma-Aldrich). The cell cycle distribution was analyzed on a flow cytometer (Beckman-Coulter). The percentages of cells in G0/G1, S and G2/M phases were determined and compared among the groups. The experiments were performed at least three times.

### Bioinformatics analysis and dual luciferase reporter assay

Target mRNAs for miR-219-5p were predicted using TargetScan (http://www.targetscan.org/) and PicTar (http://pictar.mdc-berlin.de/). The 3’UTR sequence of CCNA2 containing the predicted binding site for miR-219-5p was obtained and cloned into psiCHECK-2 vector (Promega) to give the wild-type reporter plasmid CCNA2 3’UTR-WT. To generate the CCNA2 mutant reporter plasmid, CCNA2 3’UTR-MUT, the seed region was mutated to remove all complementary nucleotides to miR-219-5p. All constructs were verified via DNA sequencing.

For the luciferase reporter assay, 293 T cells were co-transfected with 0.5 μg CCNA2 3’UTR-WT or CCNA2 3’UTR-MUT and 50 nmol miR-219-5p or miR-NC. After 48 h of transfection, luciferase activity was determined using a dual-luciferase reporter assay system (Promega) according to the manufacturer’s instructions.

### Western blotting analysis

After 48 h of cell transfection, total proteins were extracted with RIPA lysis buffer (Beyotime Biotechnology). The protein concentration was determined using a BCA protein assay kit (Beyotime Biotechnology). A total of 30 μg proteins were separated using 10% sodium dodecyl sulfate polyacrylamide gel electrophoresis (SDS-PAGE) and transferred to polyvinylidene fluoride (PVDF) membranes (Bio-Rad Laboratories).

The membranes were blocked with 5% non-fat milk in TBS containing 0.05% Tween-20 (TBST) for 1 h at room temperature, followed by incubation at 4 °C overnight with primary antibodies against cyclin A2 (Abcam) and GADPH (Santa Cruz Biotechnology, Inc.). The membranes were then washed with TBST three times, and probed with the corresponding horseradish peroxidase-conjugated secondary antibody (Santa Cruz Biotechnology, Inc.) for 2 h at room temperature. The protein signals of the membranes were visualized using an enhanced chemiluminescence reagent (Pierce).

### Statistical analysis

All the quantitative data are given as the means ± standard deviation (SD) based on at least 3 repeated experiments. SPSS 13.0 software was used for the statistical analysis. Student’s t-test was used to analyze the difference between two groups. The differences between multiple groups were calculated using one-way ANOVA with a post-hoc test. Spearman’s correlation analysis was performed to evaluate the relationship between miR-219-5p expression and CCNA2 expression and calculate coefficient (r) and *p* values. *p* < 0.05 was considered statistically significant.

## Results

### MiR-219-5p was aberrantly downregulated in ESCC tissues and cell lines

**Fig. 1:**
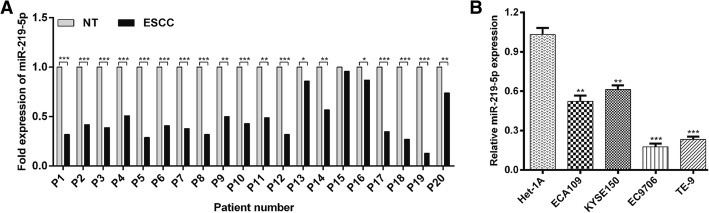
Expression levels of miR-219-5p were downregulated in ESCC tissues and cell lines. **a** A comparison of miR-219-5p expression levels between 20 paired ESCC tissues (P1–P20) and corresponding non-tumor (NT) tissues. MiR-219-5p expression was normalized to U6, calculated using the 2^-ΔΔCt^ method, and then compared with miR-219-5p expression in NT tissues. **b** Quantitative analysis of miR-219-5p levels determined using real-time PCR in four ESCC cell lines (ECA109, KYSE150, EC9706, TE-9) and one normal esophageal cell line (Het-1A). The data are presented as the means ± standard deviations. Experiments were performed in triplicate and repeated three times. *p < 0.05, **p < 0.01, ***p < 0.001; miR, microRNA; ESCC, esophageal squamous cell carcinoma

To explore the biological role of miR-219-5p in ESCC, the expression of miR-219-5p was first determined in 20 pairs of ESCC tissues and their adjacent normal tissues using real-time PCR. The expression levels of miR-219-5p were significantly decreased in most ESCC tissues in comparison with those in adjacent non-cancerous esophageal tissues (Fig. 1a).

We also evaluated the expression levels of miR-219-5p in four ESCC cell lines using real-time PCR. All the ESCC cell lines were found to have lower miR-219-5p expression levels than the normal esophageal epithelial cell line (Het-1A), with EC9706 and TE-9 cells exhibiting the lowest miR-219-5p expressions (Fig. 1b). These data suggest that miR-219-5p might play an important role in the progression of ESCC.

### Ectopic overexpression of miR-219-5p inhibited proliferation and induced G2/M arrest in ESCC cells

**Fig. 2:**
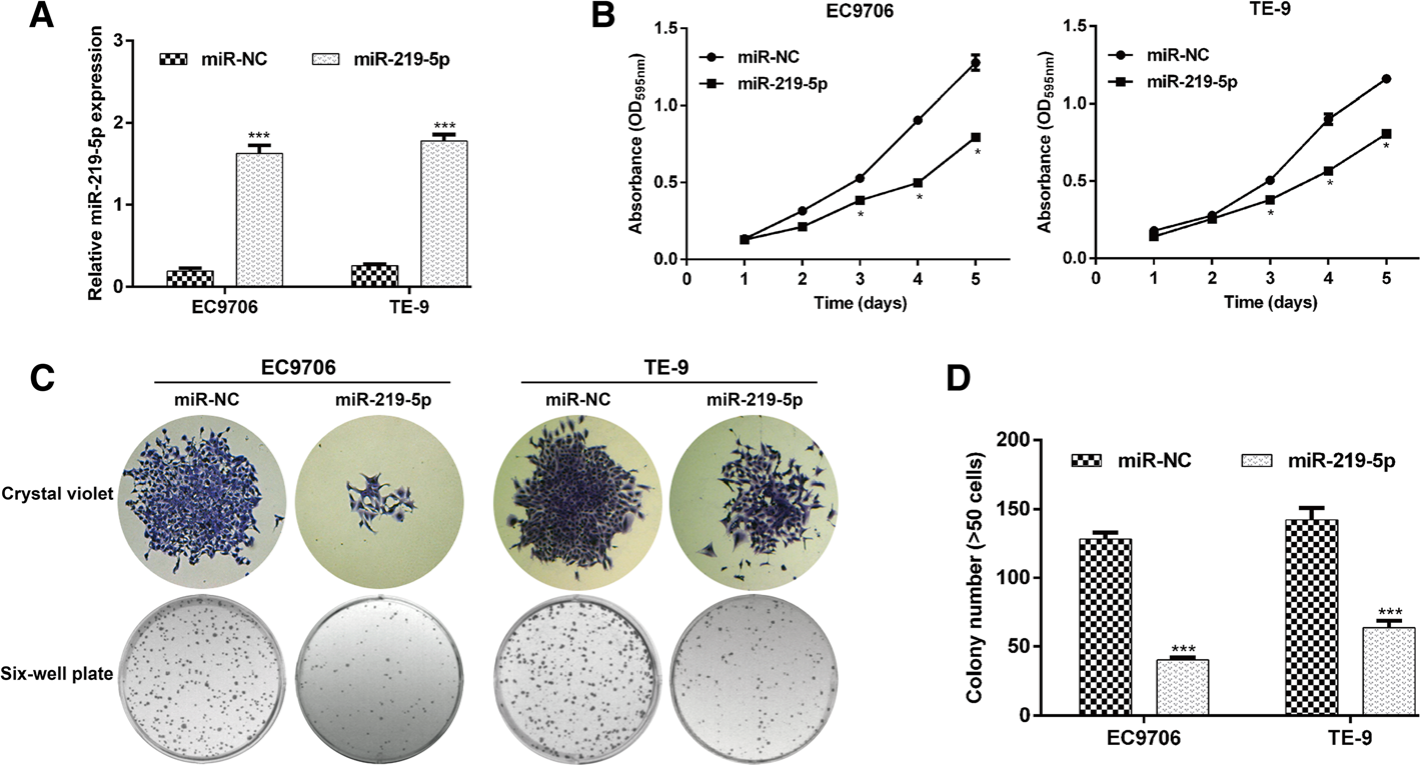
Upregulation of miR-219-5p expression inhibited cell proliferation and colon formation in ESCC. **a** The expression levels of miR-219-5p in EC9706 and TE-9 cells transfected with miR-219-5p or miR-NC were analyzed using real-time PCR. **b** Proliferation ability was tested using the MTT method in EC9706 and TE-9 cells transfected with miR-219-5p or miR-NC. **c** Representative micrographs of colony formation. **d** Relative quantification of the colonies of the indicated cells. The data are presented as means ± SD of 3 independent experiments. *p < 0.05, **p < 0.01, ***p < 0.001, compared with miR-NC

EC9706 and TE-9 cell lines with lower miR-219-5p expression were selected to study the biological function of miR-219-5p in ESCC progression in vitro. Transfection with miR-219-5p mimics enabled the establishment of lines stably overexpressing miR-219-5p. Significantly increased miR-219-5p expression after miR-219-5p transfection was confirmed using real-time PCR in both EC9706 and TE-9 cells (Fig. 2a, p < 0.001).

Next, we evaluated the effect of miR-219-5p overexpression on cell proliferation using the MTT assay. The growth curves indicate that miR-219-5p overexpression significantly suppressed the proliferation of both EC9706 and TE-9 cells (Fig. 2b, p < 0.05). The colony formation assay showed that fewer colonies were observed in EC9706 and TE-9 cells overexpressing miR-219-5p compared with control cells (Fig. 2c, p < 0.001).

**Fig. 3:**
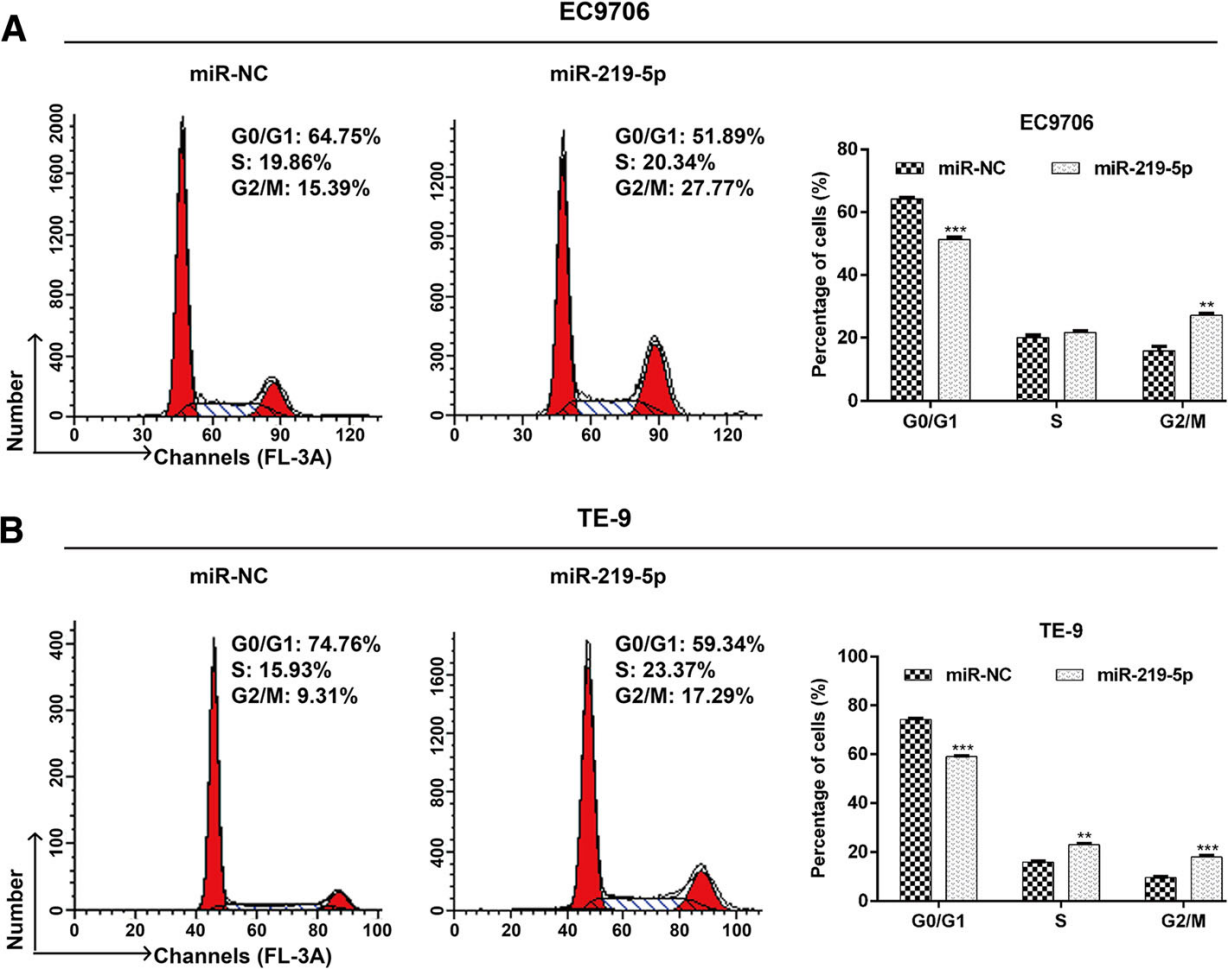
Upregulation of miR-219-5p expression induces cell cycle arrest in the G2/M phase in ESCC cells. Flow-cytometric determination of the proportion of EC9706 (**a**) and TE-9 (**b**) cells in each cell cycle phase after transfected with miR-219-5p or miR-NC. The data are presented as means ± SD of 3 independent experiments. **p < 0.01, ***p < 0.001, compared with miR-NC

To further investigate whether miR-219-5p-mediated inhibition of cell proliferation was due to cell cycle arrest, a flow cytometry assay was performed to analyze cell cycle distribution in EC9706 and TE-9 cells. The miR-219-5p overexpression resulted in a decrease in the G0/G1 phase fraction (*p* < 0.001) and an increase in G2/M phase fraction (*p* < 0.01) in EC9706 cells (Fig. 3a). Consistent results were also obtained for TE-9 cells (Fig. 3b, p < 0.01, p < 0.001). These data suggest that miR-219-5p upregulation might suppress ESCC cell proliferation partially through promotion of cell cycle G2/M phase arrest.

### MiR-219-5p downregulated CCNA2 expression by directly targeting its 3’UTR in ESCC cells

**Fig. 4:**
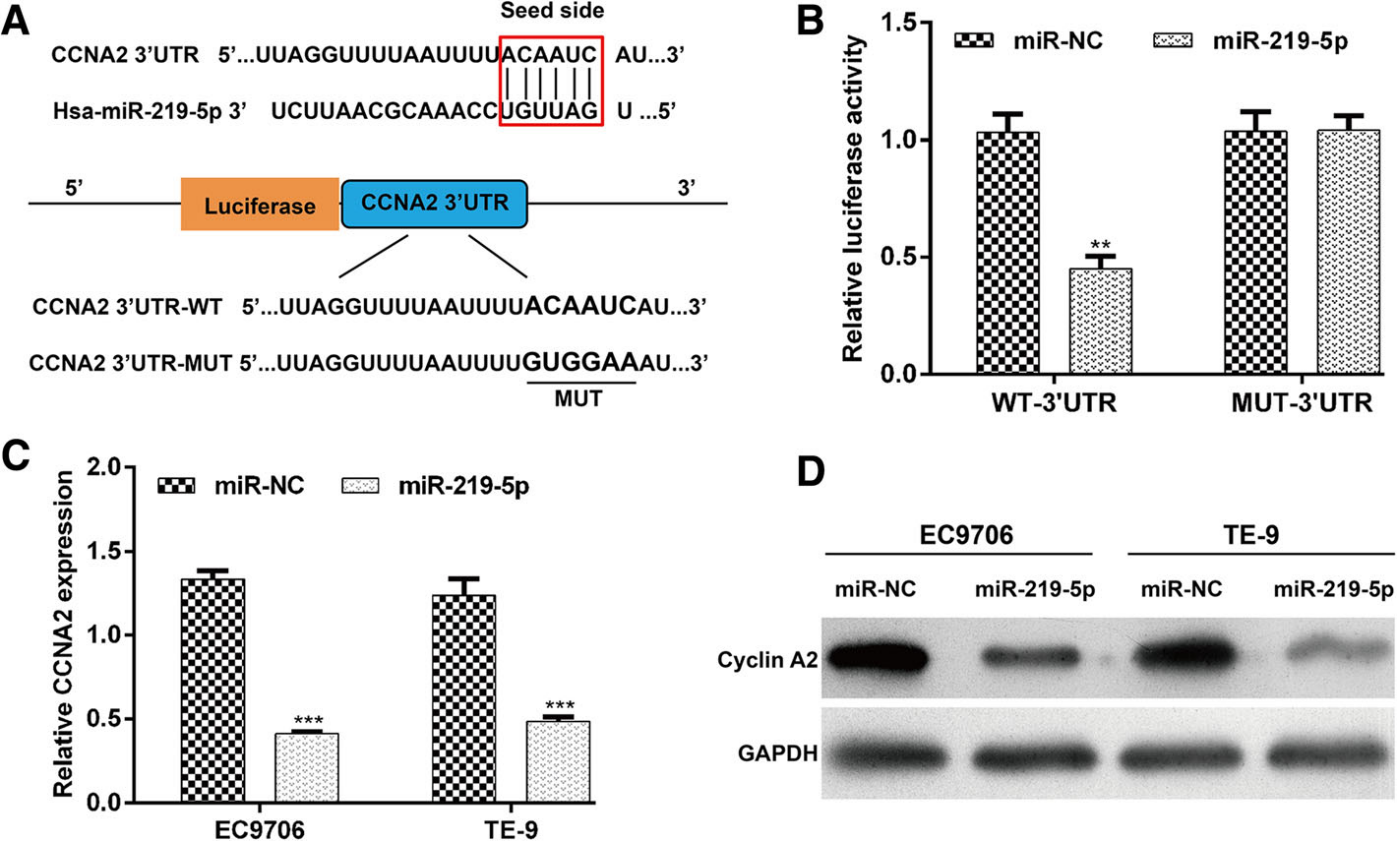
MiR-219-5p downregulated CCNA2 by directly targeting its 3’UTR. **a** Prediction of the target genes of miR-219-5p using online miRNA target prediction databases. Based on the list of the putative miR-219-5p binding sequences in the 3’-UTR of CCNA2, mutations were generated in the CCNA2 3’-UTR sequence in complementary sites for seed regions in miR-219-5p. **b** A human CCNA2 3’-UTR fragment containing either the wild-type or mutant miR-219-5p binding sequence was cloned downstream of the luciferase reporter gene. The luciferase activity was analyzed in 293 T cells. EC9706 and TE-9 cells were transfected with miR-219-5p or miR-NC and incubated for 48 h. The mRNA and protein levels of HMGA2 were measured using (**c**) real-time PCR and (**d**) western blotting. The data are presented as means ± SD of 3 independent experiments. **p < 0.01, ***p < 0.001, compared with miR-NC

The results above suggest that miR-219-5p might function as a tumor suppressor in ESCC. We further explored the functional mechanisms of miR-219-5p by investigating its downstream target genes. Online databases were used to predict its candidate target genes. CCNA2 gained our attention because it is a well-known regulator in cell cycle progression and reported to be involved in tumor progression. Putative binding sites for miR-219-5p were found in the 3’UTR of CCNA2 at 83–89 bps, which is highly conserved across species (Fig. 4a).

A luciferase reporter assay was performed to confirm CCNA2 as a direct target of miR-219-5p. The results show that overexpression of miR-219-5p significantly reduced the relative luciferase activity of the CCNA2 3’UTR WT plasmid, but did not change the luciferase activity of the CCNA2 3’UTR MUT plasmid (Fig. 4b, p < 0.01). This supports the prediction that miR-219-5p directly regulated CCNA2 by binding to its 3’UTR.

We then investigated the effects of miR-219-5p on the expression of CCNA2 in ESCC cells. The expression of CCNA2 mRNA significantly decreased in EC9706 and TE-9 cells transfected with miR-219-5p mimics (Fig. 4c, p < 0.001). Western blotting analysis further demonstrated that cyclin A2 expression was reduced at the protein level with the overexpression of miR-219-5p (Fig. 4d). These results indicate that CCNA2 is a potential target gene of miR-219-5p and could be downregulated by miR-219-5p overexpression.

### MiR-219-5p expression was inversely correlated with CCNA2 expression in ESCC

**Fig. 5:**
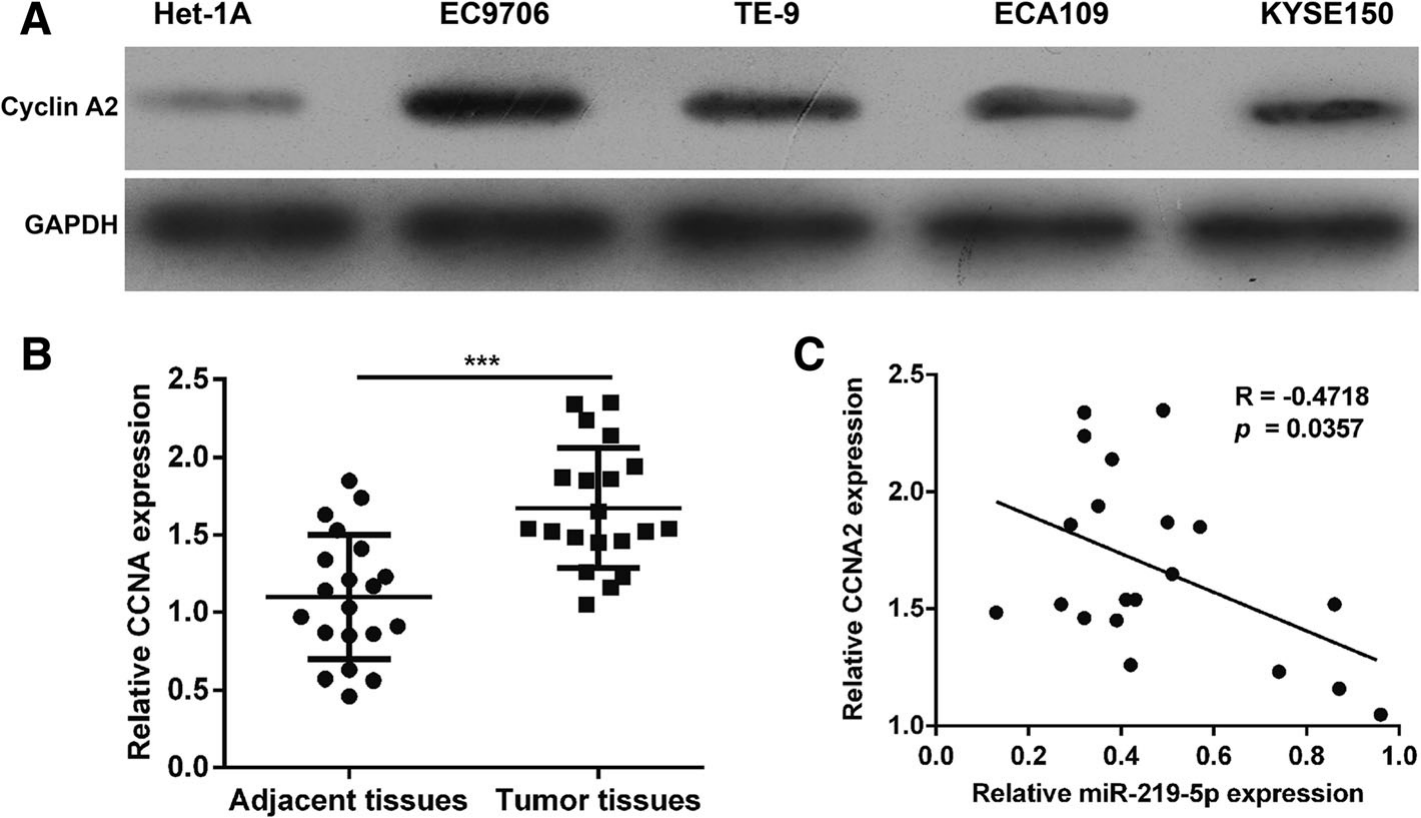
MiR-219-5p expression was inversely correlated with CCNA2 expression in ESCC. **a** Western blotting analysis of endogenous cyclin A2 expression in four ESCC and Het-1A cell lines. **b** Real-time PCR analysis of CCNA2 expression in 20 pairs of ESCC tissue samples. **c** Pearson’s correlation analysis of the relative expression levels of miR-219-5p and the relative CCNA2 mRNA levels in ESCC tissues. ***p < 0.001

We evaluated the expressions of CCNA at the protein level in the four ESCC cell lines using western blotting. Our results show obviously increased expression of cyclin A2 in all four ESCC cell lines compared with the levels for Het-1A cells. This is opposite to the downregulation of miR-219-5p expression observed in ESCC cell lines (Fig. 5a).

The expressions of CCNA2 at the mRNA level were also determined in 20 pairs of ESCC tissues. Our real-time PCR results reveal that CCNA2 expression was significantly upregulated in ESCC tissues compared with the adjacent non-tumor tissues (Fig. 5b, p < 0.001). Pearson’s correlation analysis was performed to confirm the relationship between CCNA2 and miR-219-5p expression in ESCC tissues (Fig. 5c). There was a significant negative correlation between CCNA2 expression and miR-219-5p expression in ESCC tumor samples (*p* = 0.0357, *r* = − 0.4718). From these data, we predicted that CCNA2 might be negatively regulated by miR-219-5p.

### Knockdown of CCNA2 potentiated the anti-tumor effects of miR-219-5p

**Fig. 6:**
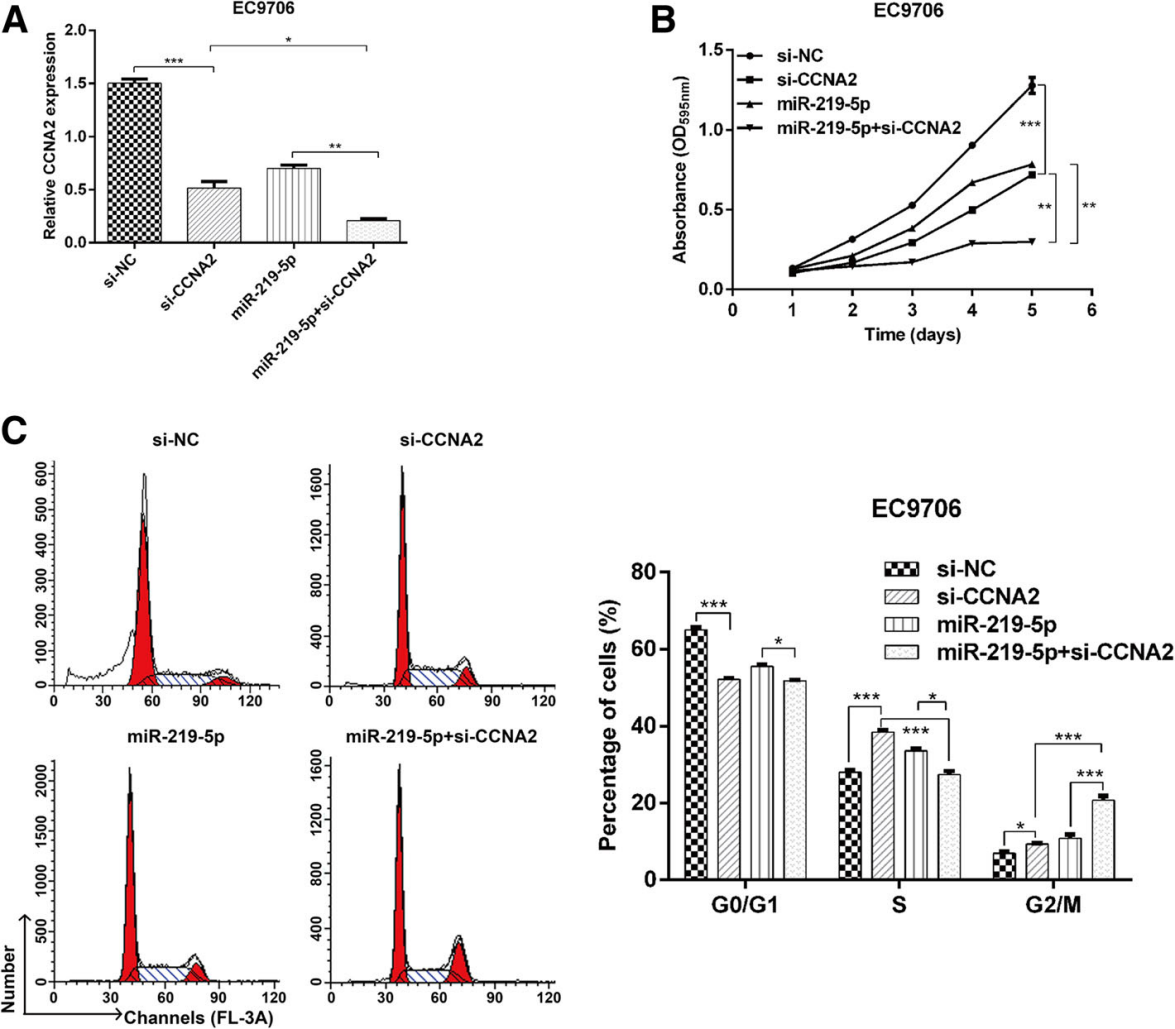
Knockdown of CCNA2 potentiated the anti-tumor effects of miR-219-5p. EC9706 cells were transfected with si-CCNA2, miR-219-5p or co-transfected with miR-219-5p and si-CCNA2. **a** Real-time PCR was used to determine the expression of CCNA2 in EC9706 cells. **b** Cell proliferation was measured using the MTT assay over five consecutive days. **c** The percentage of cells in the G0/G1, S and G2/M phases was analyzed using flow cytometry. The data are reported as means ± SD of 3 independent experiments. *p < 0.05, **p < 0.01, ***p < 0.001, compared with si-NC, si-CCNA2 or miR-219-5p

Since CCNA2 was demonstrated to be negatively regulated by miR-219-5p in ESCC, we investigated whether CCNA2 could potentiate the effects of miR-219-5p overexpression in ESCC cells. EC9706 cells were transfected with si-CCNA2 or miR-219-5p, or co-transfected with miR-219-5p and si-CCNA2. Real-time PCR confirmed that the expression of CCNA2 was significantly downregulated in EC9706 cells after transfection with si-CCNA2 or miR-219-5p alone (Fig. 6a, p < 0.001). By contrast, co-transfection with miR-219-5p and si-CCNA2 minimized the expression of miR-219-5p in EC9706 cells (*p* < 0.05, *p* < 0.01).

Our MTT analysis showed that the proliferation ability of EC9706 cells decreased after transfection with solely si-CCNA2 or miR-219-5p (Fig. 6b, p < 0.001), but a significant decrease was observed in co-transfected cells compared with cells with a single transfection (p < 0.01).

We used flow cytometry analysis to detect the effects of these transfections on cell cycle distribution. Knockdown of CCNA2 significantly reduced the percentage of cells in G0/G1 phase, but elevated the percentage of cells in S and G2/M phases. This was similar to the effect induced by miR-219-5p overexpression (p < 0.05, *p* < 0.001). Interestingly, we found that co-transfection with miR-219-5p and si-CCNA2 had more significant effects on cell cycle arrest at the G2/M phase than two single transfections (p < 0.05, p < 0.001). These results further demonstrated that knockdown of CCNA2 could strengthen the anti-tumor effects of miR-219-5p in ESCC cells.

## Discussion

Our results confirm that miR-219-5p expression level is lower in ESCC tissues and cell lines. ESCC cells treated with miR-219-5p mimics displayed a significant decrease in cell proliferation and colony formation. Furthermore, they arrested excessively in the G2/M phase compared to cells treated with miR-NC. This study indicates a fundamental role of miR-219-5p as a tumor suppressor in ESCC.

Yang et al. recently linked miR-219-5p to HCC development [13]. They established that miR-219-5p is upregulated in HCC tissues and cells with high metastatic potentials compared to non-cancerous liver tissues and non-metastatic cells [13].

By contrast, a variety of different types of cancer were demonstrated to have decreased levels of miR-219-5p and that it hard tumor suppressing activity in these cancers. For instance, it was previously reported that miR-219-5p is significantly downregulated in PTC tissues and that enhanced expression of miR-295-5p depressed the growth and survival of cancer cells by targeting estrogen receptor α [22]. Cheng et al. [23] revealed that a deficiency of miR-219-5p has been identified in colon cancer tissue specimens and that this miRNA exerts a tumor repressive role by targeting Sall4. Glioblastoma [24], gastric cancer [14] and malignant melanoma [11] were also found to have decreased miR-219-5p expression patterns.

Our results confirm that overexpression of miR-291-5p is a critical driver of ESCC cell proliferation and colony formation, and the best consideration of miR-291-5p as a potential therapeutic target for ESCC.

Interestingly, miR-219-5p negatively modulates several targets, including glypican-3, epidermal growth factor receptor (EGFR), estrogen α and sall4, which are all involved in tumorigenesis of HCC, glioblastoma, PTC and colon cancer [10, 22, 23, 25]. We explored the potential mechanism by which overexpression of miR-291-5p inhibited ESCC proliferation and colony formation. Based on our bioinformatics prediction, a critical cell cycle regulator, CCNA2 was chosen for luciferase reporter assay, because we observed a functional link between miR-219-5p and cell cycle arrest. CCNA2 was confirmed as a direct target of miR-219-5p. The effect of miR-219-5p on ESCC cell behavior could be potentiated by silencing CCNA2. Furthermore, miR-219-5p mimics and si-CCNA2 co-transfection had more significant effects on cell proliferation and G2/M phase arrest than two single transfections.

Under complex pathological conditions, the entry of cancer cells and their progeny into uncontrolled cell growth are driven by a limited number of “mitosis events” [26]. Cyclin family members have long been considered as critical regulators of cell cycle progression [27]. CCNA2 is a ubiquitously expressed member of the cyclin family [28, 29]. It plays an indispensable role in regulating G1/S transition and in the actual process of mitosis through the activation of kinases [29]. CCNA2 is frequently related to cell proliferation and expressed at high levels in many cancers [30]. Stage I, II, III and IV ESCC tumors exhibit stronger CCNA2 immunoreactivity than earlier phases of development [31]. High CCNA2 immunopositivity is associated with tumor development and poor survival rate of ESCC patients [31].

Our study demonstrated that knockdown of CCNA2 imitated cell cycle arrest at G2/M phase, induced by overexpression of miR-219-5p. Co-transfection with miR-219-5p mimics and si-CCNA2 showed the highest arrest rates at G2/M and the lowest proliferation capability in vitro than either transfection alone.

Interestingly, accumulating evidence indicates that G2/M arrest is linked to inhibition of cancer proliferation. Lin et al. [32] confirmed that depletion of eIF-3d reduces non-small cell lung cancer cell proliferation via enhancement of cell cycle arrest in the G2/M phase. Sun et al. [33] proved that the dendrobium candium could suppress the proliferation of MCF-7 cells by increasing G2/M arrest. Here, we suggest that miR-219-5p suppressed ESCC cell proliferation and induced G2/M phase arrest by directly downregulating CCNA2 expression.

## Conclusions

Our data show that miR-219-5p is downregulated in ESCC tissues and cell lines and prevents ESCC cells expansion by negatively regulating CCNA2. The newly identified role of miR-219-5p in ESCC cells may provide a potential therapeutic strategy for cancer drug discovery.

## Abbreviations

CCNA2: cyclin A2
CDKs: cyclin-dependent kinases
ESCC: esophageal squamous cell carcinoma

## References

[1] Xu Y, Wang J, Qiu M, Xu L, Li M, Jiang F, Yin R, Xu L (2015). Upregulation of the long noncoding RNA TUG1 promotes proliferation and migration of esophageal squamous cell carcinoma. Tumour Biol.

[2] Hu L, Wu Y, Tan D, Meng H, Wang K, Bai Y, Yang K (2015). Up-regulation of long noncoding RNA MALAT1 contributes to proliferation and metastasis in esophageal squamous cell carcinoma. J Exp Clin Cancer Res.

[3] Sawada G, Niida A, Uchi R, Hirata H, Shimamura T, Suzuki Y, Shiraishi Y, Chiba K, Imoto S, Takahashi Y (2016). Genomic landscape of esophageal squamous cell carcinoma in a Japanese population. Gastroenterology.

[4] Ohashi S, Miyamoto S, Kikuchi O, Goto T, Amanuma Y, Muto M (2015). Recent advances from basic and clinical studies of esophageal squamous cell carcinoma. Gastroenterology.

[5] Agrawal N, Jiao Y, Bettegowda C, Hutfless SM, Wang Y, David S, Cheng Y, Twaddell WS, Latt NL, Shin EJ (2012). Comparative genomic analysis of esophageal adenocarcinoma and squamous cell carcinoma. Cancer Discov.

[6] Lin DC, Hao JJ, Nagata Y, Xu L, Shang L, Meng X, Sato Y, Okuno Y, Varela AM, Ding LW (2014). Genomic and molecular characterization of esophageal squamous cell carcinoma. Nat Genet.

[7] Piletic K, Kunej T (2016). MicroRNA epigenetic signatures in human disease. Arch Toxicol.

[8] HafezQorani S, Lafzi A, de Bruin RG, van Zonneveld AJ, van der Veer EP, Son YA, Kazan H (2016). Modeling the combined effect of RNA-binding proteins and microRNAs in post-transcriptional regulation. Nucleic Acids Res.

[9] Oliveto S, Mancino M, Manfrini N, Biffo S (2017). Role of microRNAs in translation regulation and cancer. World J Biol Chem.

[10] Rao SA, Arimappamagan A, Pandey P, Santosh V, Hegde AS, Chandramouli BA, Somasundaram K (2013). miR-219-5p inhibits receptor tyrosine kinase pathway by targeting EGFR in glioblastoma. PLoS One.

[11] Long J, Menggen Q, Wuren Q, Shi Q, Pi X (2017). MiR-219-5p inhibits the growth and metastasis of malignant melanoma by targeting BCL-2. Biomed Res Int.

[12] Xiong GB, Zhang GN, Xiao Y, Niu BZ, Qiu HZ, Wu B, Lin GL, You L, Shu H (2015). MicroRNA-219-5p functions as a tumor suppressor partially by targeting platelet-derived growth factor receptor alpha in colorectal cancer. Neoplasma.

[13] Yang J, Sheng YY, Wei JW, Gao XM, Zhu Y, Jia HL, Dong QZ, Qin LX (2018). MicroRNA-219-5p promotes tumor growth and metastasis of hepatocellular carcinoma by regulating cadherin 1. Biomed Res Int.

[14] Li C, Dong J, Han Z, Zhang K (2017). MicroRNA-219-5p represses the proliferation, migration, and invasion of gastric Cancer cells by targeting the LRH-1/Wnt/beta-catenin signaling pathway. Oncol Res.

[15] Huang LX, Hu CY, Jing L, Wang MC, Xu M, Wang J, Wang Y, Nan KJ, Wang SH (2017). microRNA-219-5p inhibits epithelial-mesenchymal transition and metastasis of colorectal cancer by targeting lymphoid enhancer-binding factor 1. Cancer Sci.

[16] Hydbring P, Malumbres M, Sicinski P (2016). Non-canonical functions of cell cycle cyclins and cyclin-dependent kinases. Nat Rev Mol Cell Biol.

[17] Tiwari S, Roel C, Wills R, Casinelli G, Tanwir M, Takane KK, Fiaschi-Taesch NM (2015). Early and late G1/S cyclins and Cdks act complementarily to enhance authentic human beta-cell proliferation and expansion. Diabetes.

[18] Otero JJ, Kalaszczynska I, Michowski W, Wong M, Gygli PE, Gokozan HN, Griveau A, Odajima J, Czeisler C, Catacutan FP (2014). Cerebellar cortical lamination and foliation require cyclin A2. Dev Biol.

[19] Krasnov GS, Puzanov GA, Kudryavtseva AV, Dmitriev AA, Beniaminov AD, Kondratieva TT, Senchenko VN (2017). Differential expression of an ensemble of the key genes involved in cell-cycle regulation in lung cancer. Mol Biol (Mosk).

[20] Zhong W-B, Hsu S-P, Ho P-Y, Liang Y-C, Chang T-C, Lee W-S (2011). Lovastatin inhibits proliferation of anaplastic thyroid cancer cells through up-regulation of p27 by interfering with the rho/ROCK-mediated pathway. Biochem Pharmacol.

[21] He Y, Liu J, Zhao Z, Zhao H (2017). Bioinformatics analysis of gene expression profiles of esophageal squamous cell carcinoma. Dis Esophagus.

[22] Huang C, Cai Z, Huang M, Mao C, Zhang Q, Lin Y, Zhang X, Tang B, Chen Y, Wang X (2015). miR-219-5p modulates cell growth of papillary thyroid carcinoma by targeting estrogen receptor alpha. J Clin Endocrinol Metab.

[23] Cheng J, Deng R, Zhang P, Wu C, Wu K, Shi L, Liu X, Bai J, Deng M, Shuai X (2015). miR-219-5p plays a tumor suppressive role in colon cancer by targeting oncogene Sall4. Oncol Rep.

[24] Jiang Y, Yin L, Jing H, Zhang H (2015). MicroRNA-219-5p exerts tumor suppressor function by targeting ROBO1 in glioblastoma. Tumour Biol.

[25] Huang N, Lin J, Ruan J, Su N, Qing R, Liu F, He B, Lv C, Zheng D, Luo R (2012). MiR-219-5p inhibits hepatocellular carcinoma cell proliferation by targeting glypican-3. FEBS Lett.

[26] Asghar U, Witkiewicz AK, Turner NC, Knudsen ES (2015). The history and future of targeting cyclin-dependent kinases in cancer therapy. Nat Rev Drug Discov.

[27] Mueller D, Totzke F, Weber T, Beisenherz-Huss C, Kraemer D, Heidemann-Dinger C, Ketterer C, Eckert C, Kubbutat (2016). MHG: Abstract 2821: characterization of CDK inhibitors in a biochemical assay using a comprehensive panel of human CDK-cyclin complexes. Cancer Res.

[28] Bendris N, Loukil A, Cheung C, Arsic N, Rebouissou C, Hipskind R, Peter M, Lemmers B, Blanchard JM (2012). Cyclin A2: a genuine cell cycle regulator?. Biomol Concepts.

[29] Loukil A, Cheung CT, Bendris N, Lemmers B, Peter M, Blanchard JM (2015). Cyclin A2: at the crossroads of cell cycle and cell invasion. World J Biol Chem.

[30] Liang W, Guan H, He X, Ke W, Xu L, Liu L, Xiao H, Li Y (2015). Down-regulation of SOSTDC1 promotes thyroid cancer cell proliferation via regulating cyclin A2 and cyclin E2. Oncotarget.

[31] Furihata M, Ishikawa T, Inoue A, Yoshikawa C, Sonobe H, Ohtsuki Y, Araki K, Ogoshi S (1996). Determination of the prognostic significance of unscheduled cyclin a overexpression in patients with esophageal squamous cell carcinoma. Clin Cancer Res.

[32] Lin Z, Xiong L, Lin Q (2015). Knockdown of eIF3d inhibits cell proliferation through G2/M phase arrest in non-small cell lung cancer. Med Oncol.

[33] Sun J, Guo Y, Fu X, Wang Y, Liu Y, Huo B, Sheng J, Hu X (2016). Dendrobium candidum inhibits MCF-7 cells proliferation by inducing cell cycle arrest at G2/M phase and regulating key biomarkers. Onco Targets Ther.

